# Activity-relevant similarity values for fingerprints and implications for similarity searching

**DOI:** 10.12688/f1000research.8357.2

**Published:** 2016-04-28

**Authors:** Swarit Jasial, Ye Hu, Martin Vogt, Jürgen Bajorath

**Affiliations:** 1 Department of Life Science Informatics, B-IT, LIMES Program Unit Chemical Biology and Medicinal Chemistry, Rheinische Friedrich-Wilhelms-Universität, Bonn, Germany

**Keywords:** Bioactive compounds, molecular similarity, similarity-property principle, similarity searching, fingerprints, Tanimoto coefficient, activity similarity

## Abstract

A largely unsolved problem in chemoinformatics is the issue of how calculated compound similarity relates to activity similarity, which is central to many applications. In general, activity relationships are predicted from calculated similarity values. However, there is no solid scientific foundation to bridge between calculated molecular and observed activity similarity. Accordingly, the success rate of identifying new active compounds by similarity searching is limited. Although various attempts have been made to establish relationships between calculated fingerprint similarity values and biological activities, none of these has yielded generally applicable rules for similarity searching. In this study, we have addressed the question of molecular versus activity similarity in a more fundamental way. First, we have evaluated if activity-relevant similarity value ranges could in principle be identified for standard fingerprints and distinguished from similarity resulting from random compound comparisons. Then, we have analyzed if activity-relevant similarity values could be used to guide typical similarity search calculations aiming to identify active compounds in databases. It was found that activity-relevant similarity values can be identified as a characteristic feature of fingerprints. However, it was also shown that such values cannot be reliably used as thresholds for practical similarity search calculations. In addition, the analysis presented herein helped to rationalize differences in fingerprint search performance.

## Introduction

Calculation of molecular similarity is a central task in chemoinformatics ^1– 4^ for which a variety of methods, chemical descriptors, and similarity measures have been introduced ^1– 7^. A key aspect of the molecular similarity concept is that one often attempts to extrapolate from calculated similarity to activity similarity. In other words, it is assumed that increasing chemical similarity correlates with an increasing likelihood that two compounds share the same activity, in accord with the similarity-property principle (“similar compounds should have similar properties”) ^1^; a major foundation of chemoinformatics. A methodological consequence of the molecular similarity concept and similarity-property principle was the introduction of similarity searching for active compounds ^2, 6^. Here, similarity values are calculated for known active reference and database compounds, which are then ranked in the order of decreasing similarity to the reference(s). Classical molecular descriptors for these search calculations include 2D-fingerprints, i.e. bit string representations of chemical structures and/or properties derived from molecular graphs ^2, 8, 9^. The overlap between fingerprints is quantified as a measure of molecular similarity using metrics such as the Tanimoto coefficient (Tc) ^2, 7^; the gold standard in the chemoinformatics field. For a pair of compounds represented by fingerprints, its Tc value is calculated as the ratio between the number of features conserved in both fingerprints and the number of features present in either fingerprint. Accordingly, the Tc is a numerical measure of similarity ranging from zero (no fingerprint overlap) to one (fingerprint identity).

Similarity search calculations exemplify the similarity conundrum in chemoinformatics: the ultimate goal is the identification of new active compounds on the basis of similarity, but activity information is not used as a search parameter. It has been shown that generally applicable Tc threshold values as an indicator of activity similarity do not exist ^9^. This is the case because similarity value distributions are compound class- and fingerprint-dependent. To further complicate matters, it has also been shown that 2D-fingerprints successfully detect structurally diverse active compounds at varying similarity levels ^9, 10^. In general, a continuum of similarity values is produced that may or may not indicate activity similarity, depending on the characteristics of active compounds.

A limited number of attempts have been made to associate calculated similarity with observed activity similarity. For example, early investigations of compound clustering, molecular diversity, and chemical neighborhood behavior using fingerprints have indicated that, on average, 85% of compounds that yielded a Tc value of 0.85 compared to a known active molecule were also active ^11– 13^. These findings were based on MACCS keys ^14^, a classical fingerprint in chemoinformatics consisting of a dictionary of 166 structural fragments, as well as UNITY fingerprints ^13^ that assemble atom pathways of pre-defined lengths. However, using connectivity pathway fingerprints of different design and biological screening data to analyze the relationship between calculated similarity and observed activity similarity, it was concluded that there was only a likelihood of 30% that compounds yielding a Tc value of at least 0.85 shared the same activity ^15^. In addition, Kullback-Leibler divergence analysis from information theory and Bayesian modeling were combined ^16^ to predict the recall of active compounds from fingerprint similarity searching and a conditional correlated Bernoulli model of similarity value distributions was developed ^17^ to predict database rankings. Furthermore, belief theory was applied to empirically derive probabilistic relationships between calculated similarity and activity on the basis of similarity search benchmark calculations ^18^. In this study, MACCS keys and extended connectivity fingerprints (ECFPs) ^19^ were used, among others. ECFPs capture layered atom environments in compounds up to a pre-determined bond diameter. When different fingerprints were compared in benchmark calculations ECFPs often yielded highest similarity search performance ^8, 9^. On the basis of probability assignment curves that related activity and similarity values for pairs of compounds to each other, it was shown, for example, that at a Tc value of 0.85 calculated with atom pathway fingerprints, ~30% of detected compound pairs shared the same activity ^18^, consistent with earlier observations ^15^. For an ECFP with bond diameter 6 (ECFP6), a Tc threshold of 0.42 yielded comparable results ^18^.

Other types of fingerprints were generated exclusively on the basis of experimental activity observations, e.g. activities measured in panels of screening assays ^20^, or by combining chemical and biological criteria ^21^. These studies departed from the conceptual framework of the similarity-property principle by using activity data as descriptors and -completely or partly- circumventing similarity calculations on the basis of molecular structures.

Herein, we report an analysis designed to rationalize similarity searching on the basis of different molecular comparisons, carried out on a large scale, and determine similarity values across different compound activity classes. It is shown that similarity value ranges indicative of activity can be identified for different fingerprints. However, it is also shown that such similarity values cannot be reliably used as thresholds for similarity searching, given the ratio of different molecular comparisons that are involved.

## Materials and methods

### Compound classes

In a previous large-scale similarity search analysis of the ChEMBL database ^22^, a variety of activity classes were identified for benchmarking that were “easy” (i.e. yielded generally high compound recall using different fingerprints), “preferred/intermediate” (moderate compound recall), or “difficult” (low compound recall) ^23^. For our analysis, we have made use of this classification scheme and extracted these activity classes from ChEMBL version 20 if they contained at least 50 compounds with high-confidence assay data for human targets ^24^ and a potency of at least 10 µM. It should be noted that fingerprint searching does not take activity as a parameter into account. Therefore, the potency threshold value was only applied to exclude compounds from the calculations whose activity would be considered borderline, despite the presence of high-confidence activity data. In addition, a random sample of 10,000 compounds was drawn from ZINC ^25^ representing assumed inactive database compounds. All randomly selected (“random”) compounds had a molecular weight of less than 550 Da. Accordingly, all compounds with a molecular weight exceeding 550 Da were also removed from activity classes, thus balancing the potential of molecular size effects in similarity searching ^26^.

**Figure 1.**
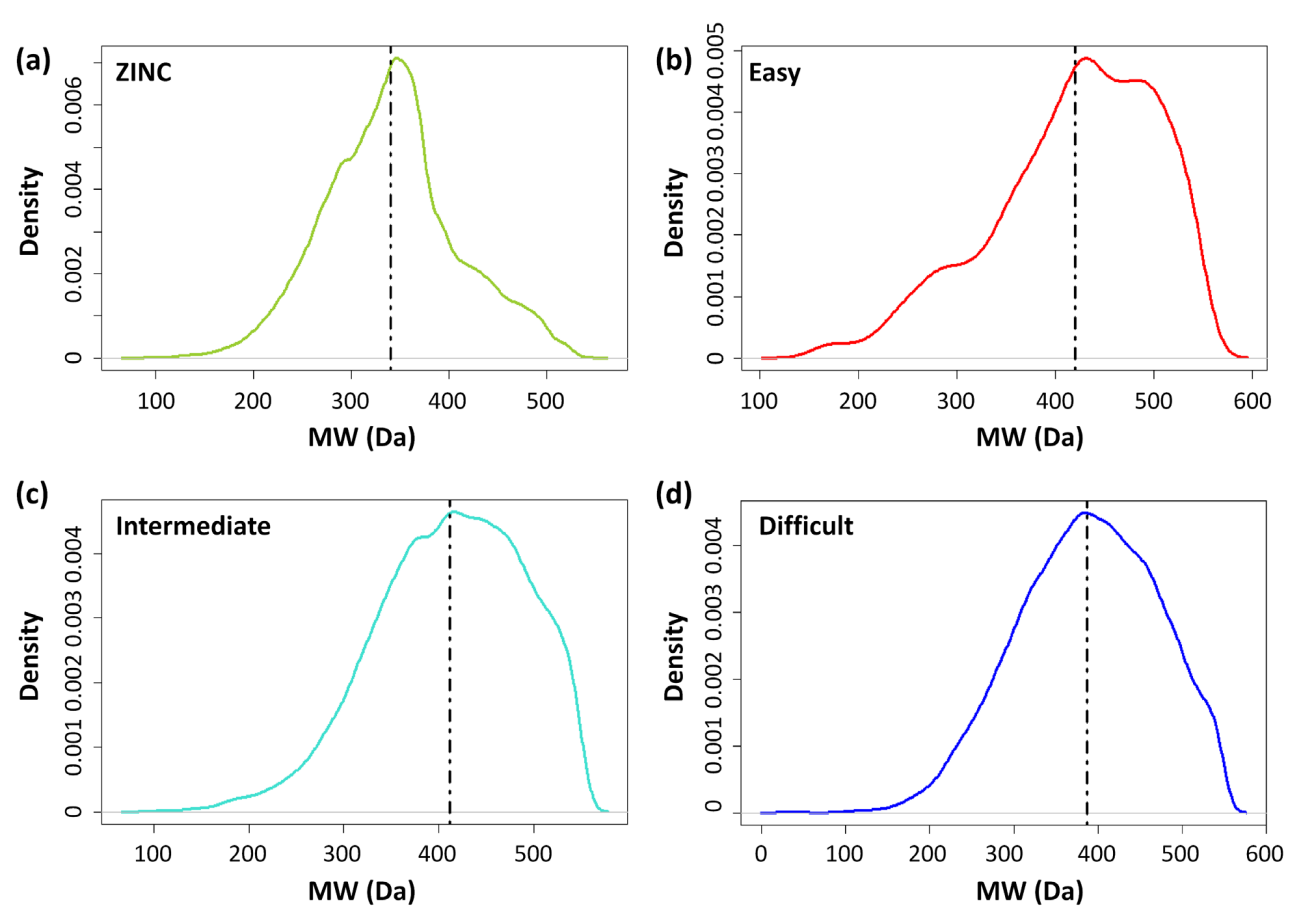
Molecular weight ranges. Density plots report the molecular weight (Da) distributions of compounds in all categories.

On the basis of these criteria, 22 easy, 50 intermediate, and 30 difficult activity classes were obtained covering a wide range of targets. Easy activity classes contained a total of 2967 compounds with, on average, 135 compounds per target; intermediate activity classes contained 25,175 compounds with a mean of 504 per target, and difficult activity classes 47,109 compounds with a mean of 1570 per target. The molecular weight distributions of compounds from all categories are reported in Figure 1. Compounds from ZINC had overall slightly lower weight than active compounds but the distributions of molecular weights of from different activity class categories were very similar.

### Similarity calculations

Two standard fingerprints of different design were used including MACCS and ECFP with bond diameter 4 (ECFP4). These fingerprint representations were calculated using an in-house script. For MACCS, settings from RDKit ^27^ were used. For ECFP4, the original design was re-implemented ^19^. As a similarity metric, the Tc was calculated.

Systematic pairwise similarity calculations were carried out for all individual activity classes (active vs. active), the random category (random vs. random), and active vs. random compounds.

## Results and discussion

### Comparison of compounds belonging to different categories

During similarity searching, active reference compounds are compared to inactive database compounds or desired compounds having the same activity (“hits”). Thus, similarity searching can be mimicked by systematically comparing active compounds having the same activity with each other and active compounds to random database compounds. Comparison of random database compounds with each other is not carried out during traditional similarity searching, but the similarity value distribution resulting from this comparison can be monitored as an additional reference.

### Distribution of combined similarity values

**Figure 2.**
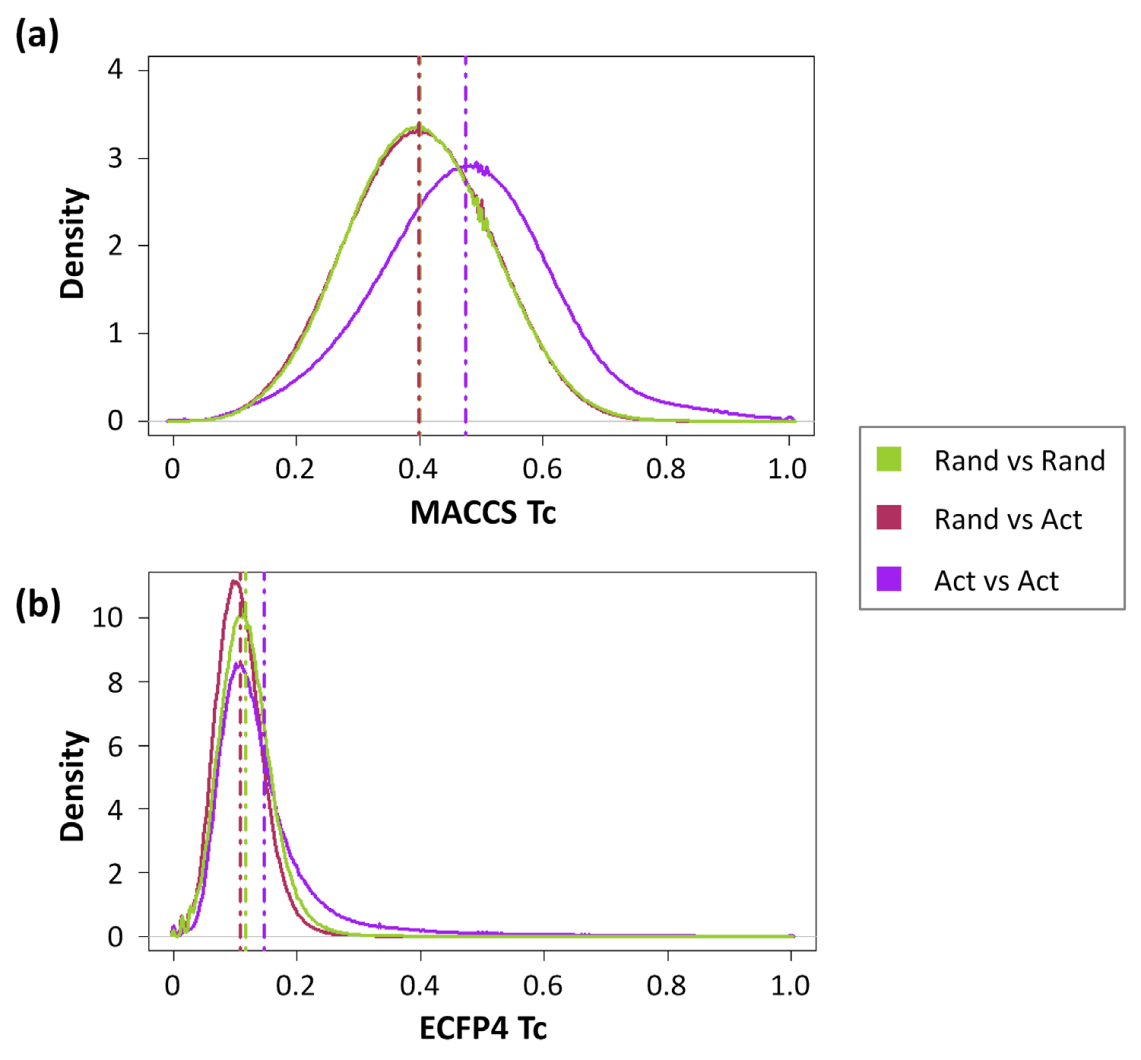
Distribution of combined similarity values. Density plots of Tc values are shown for similarity comparison using ( **a**) MACCS and ( **b**) ECFP4. Compared were active compounds in each activity class (Act vs Act, purple), 10,000 random ZINC compounds with each activity class (Rand vs Act, maroon), and 10,000 random compounds (Rand vs Rand, green). Similarity values of all 102 activity classes were combined. Dashed vertical lines indicate the means of the distributions.

Figure 2 shows the distribution of Tc values from active vs. active, random vs. active, and random vs. random compound comparisons using MACCS and ECFP4. For this comparison, Tc values obtained for all activity classes were combined. Thus, the resulting distribution represented ~55 million Tc values for compounds active against 102 targets. Comparison of ZINC compounds yielded 50 million Tc values. Their distribution was regarded to represent global chemical similarity (although many ZINC compounds are considered “drug-like”) and thus termed “chemical similarity distribution”.

Figure 2a shows that global similarity values calculated using MACCS yielded a normal distribution, given its very large sample size, which was centered on a MACCS Tc value of 0.4. The comparison of random vs. active compounds using MACCS, resulting in a total of ~753 million Tc values, 50 million of which were randomly sampled for the generation of density plots, produced a nearly identical normal distribution, also reflecting randomness. By contrast, the distribution of Tc values from active compounds, albeit significantly overlapping with the reference distributions, was shifted to the right, centered on a MACCS Tc value of 0.47. This distribution was regarded to represent activity-relevant similarity, given that it originated from more than 100 qualifying activity classes.

Figure 2b shows that calculations using ECFP4 produced very different Tc value distributions. Compared to MACCS, ECFP4 Tc value distributions were shifted towards much lower Tc values and confined to small value ranges mostly falling within the interval [0.0, 0.2] (which should be known to similarity search practitioners). The chemical similarity distribution and random vs. active distribution were centered on an ECFP4 Tc value of 0.11. Also in this case, a slight shift of the activity-relevant distribution towards higher values was observed, centered on an ECFP4 Tc of 0.15. Hence, for both ZINC and ChEMBL compounds, ECFP4 calculations mostly covered only small Tc value ranges.

**Figure 3.**
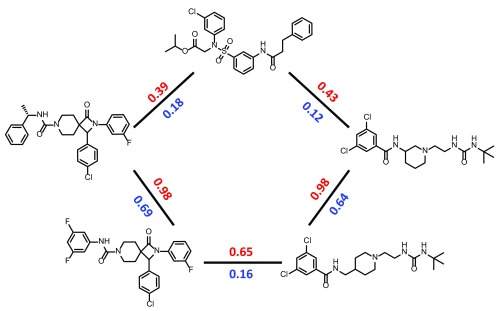
Pairs of active compounds with varying similarity values. Shown are five exemplary compounds active against the voltage-gated T-type calcium channel alpha-1H subunit (an “easy” activity class). Five pairwise similarity values calculated using MACCS (red) and ECFP4 (blue), respectively, are reported.

It should also be noted that the distribution of similarity values of compounds sharing the same activity often covered a wide range, as illustrated in Figure 3. Thus, many activity classes were structurally diverse.

### Distributions for different activity class categories

**Figure 4.**
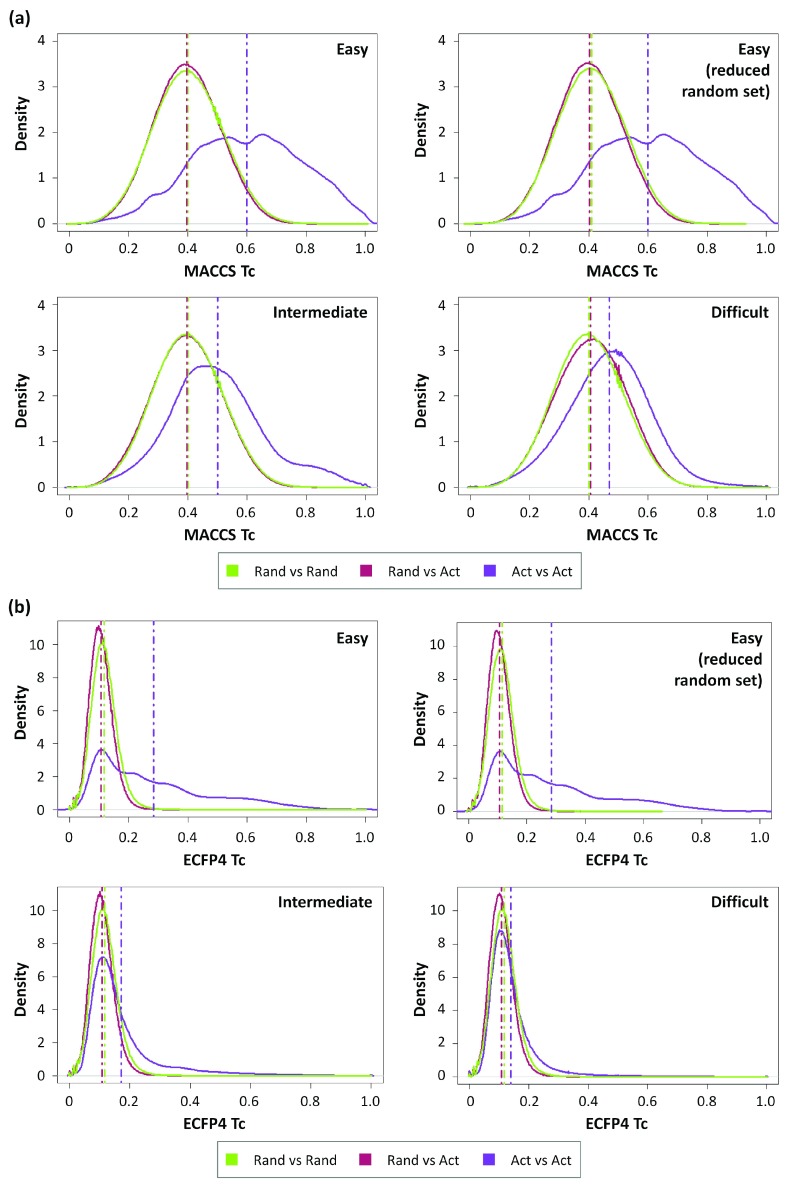
Distribution of similarity values for different activity class categories. Density plots of Tc values are shown for similarity comparison using ( **a**) MACCS and ( **b**) ECFP4 according to Figure 2. In this case, Tc value distributions were separately recorded for each activity class category (easy, intermediate, and difficult). In addition, easy activity classes were compared to a reduced random set of 1000 ZINC compounds (reported in the upper right panels).

Figure 4 shows corresponding Tc value distributions that were separately generated for easy, intermediate, and difficult activity classes. Comparison of these value distributions nicely correlated with the different similarity search performance observed for these activity classes.

In Figure 4a, the chemical similarity and random vs. active distributions were again essentially identical and centered on a MACCS Tc value of 0.4, although the sample sizes of active compounds were smaller in this case. The random vs. active distribution did not change when the random sample was reduced in size from 10,000 to 1000 ZINC compounds, indicating that the distribution was stable. Equivalent observations were made for the distributions of ECFP4 values shown in Figure 4b **.**

However, for both MACCS and ECFP4, gradual shifts in the distributions of Tc values for different activity class categories were observed. From difficult over intermediate to easy activity classes, the activity-relevant distributions shifted towards higher Tc values. Thus, corresponding to increasing similarity search performance, the comparison of active compounds produced higher Tc values than random vs. active comparisons, leading to an enrichment of active compounds at higher positions in similarity-based rankings. For easy activity classes, the shape of the distributions departed from normal distributions and became multi-modal, probably reflecting activity class-dependent differences in Tc values. These distributions displayed a significant shift towards higher Tc values with a mean of 0.6 and 0.28 for MACCS and ECFP4, respectively.

### Activity-relevant similarity

**Figure 5.**
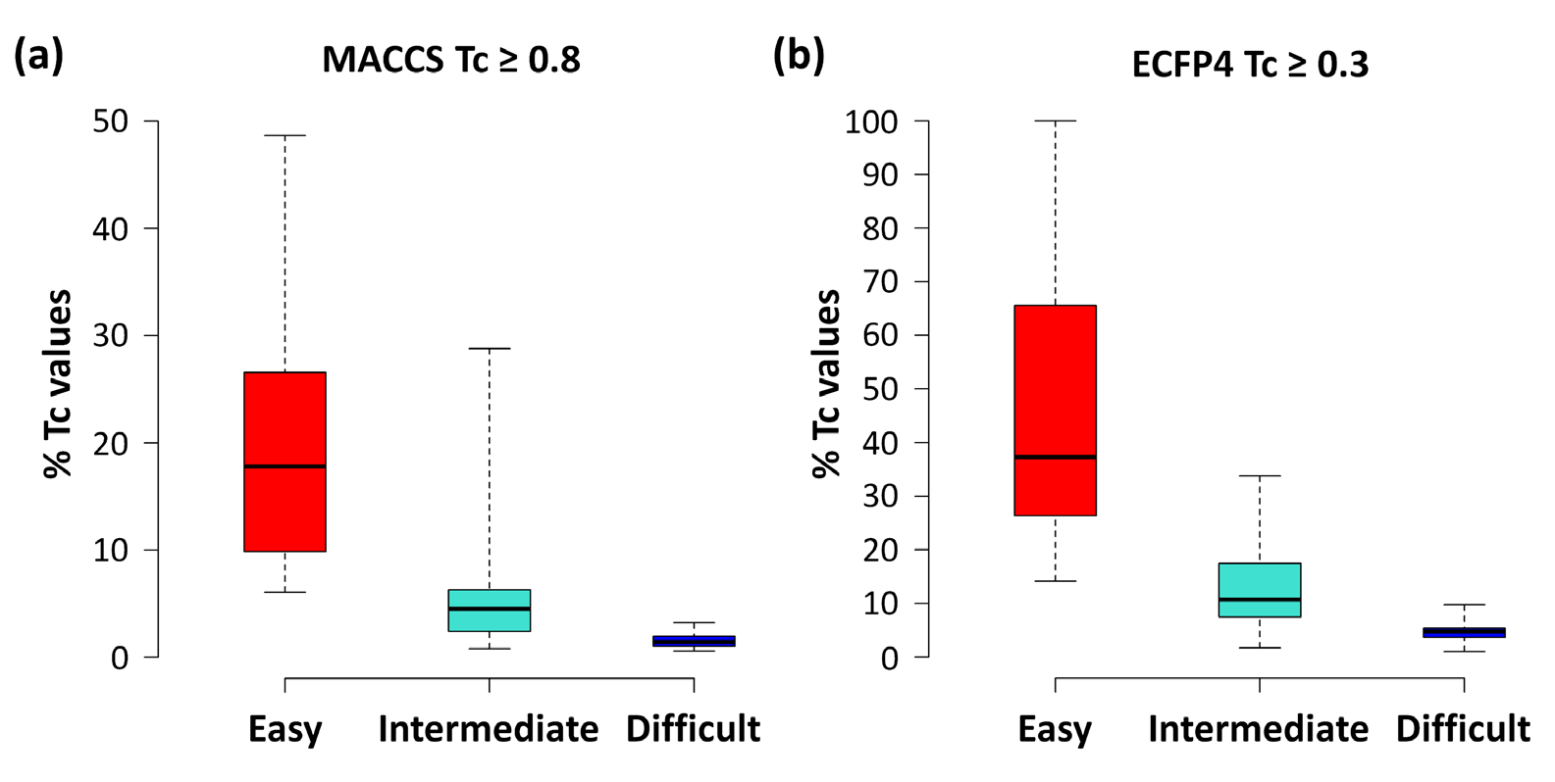
Distribution of similarity values in activity-relevant ranges. Box plots report the distribution of the percentage of Tc values falling into activity-relevant ranges for each category of activity classes using ( **a**) MACCS and ( **b**) ECFP4. A box plot gives the minimum percentage of Tc values in the activity-relevant range per category (bottom line), first quartile (lower boundary of the box), median value (thick line), third quartile (upper boundary of the box), and highest percentage of Tc values (top line).

Comparison of the distributions in Figure 4 made it possible to delineate activity-relevant similarity value ranges. In Figure 4a, the chemical similarity and random vs. active distributions for MACCS matched the baseline at a value of ~0.8, whereas a significant proportion of Tc values of 0.8 or greater were observed for comparisons of active compounds, especially for easy activity classes. Equivalent observations were made for an ECFP4 Tc value of ~0.3 shown in Figure 4b. Thus, for MACCS and ECFP4, there was a much higher probability that comparison of active compounds yielded a Tc value of at least 0.8 and 0.3, respectively, than comparison of active vs. random (or random vs. random) compounds. Figure 5 reports for all activity class categories the percentages of Tc values of at least 0.8 (MACCS, Figure 5a) and 0.3 (ECFP4, Figure 5b). These percentages significantly increased for difficult over intermediate to easy activity classes, (again mirroring similarity search performance), reaching medians of 17.7% (MACCS) and 37.5% (ECFP4), with a significant spread of percentages among easy activity classes, as revealed by the box plot representations in Figure 5.

Taken together, these findings show that it was possible to detect activity-relevant Tc value ranges for different fingerprints by systematically comparing Tc value distributions for active and randomly selected compounds.

### Implications for similarity searching

A key question was whether activity-relevant similarity values might also serve as threshold values for similarity searching, contrary to the conclusions drawn from earlier studies analyzing compound recall rates and rankings. In this context, the ratio of different compound comparisons involved in similarity search calculations must be considered, as discussed below. The activity-relevant Tc value ranges of ≥ 0.8 (MACCS) and ≥ 0.3 (ECFP4) derived from comparison of similarity value distributions are plausible likelihood estimates, as further supported by the data in Table 1. For example, for easy activity classes, 16% of MACCS Tc values for comparison of active compounds reached or exceeded 0.8, whereas this was only the case for 0.005% of random vs. active comparisons. For ECFP4, the corresponding percentages were 38.2% and 0.03%, respectively. However, in a typical similarity search trial, many more active vs. random compound comparisons are carried out than active vs. active comparisons, given that only small numbers of compounds with a specific activity are usually available in databases. For example, let us consider the most favorable case of easy activity classes, a search with MACCS using a single active reference compound, and a similarity threshold value of 0.8. If a database of 100,000 inactive and 50 active compounds were to be screened, eight active compounds would be detected together with five false-positives on the basis of the ratios given in Table 1. If 500,000 database compounds were to be screened, the number of false-positives would increase to 25 (given that the active vs. random distribution was normal). For ECFP4, applying a similarity threshold value of 0.3 under the same search conditions, screening a database with 50 active and 500,000 inactive compounds would result in 19 true- and 150 false-positives. Hence, even for easy activity classes, activity-relevant similarity values could not be reliably applied as thresholds in a typical similarity search scenario because of the large discrepancy in the number of different comparisons. Furthermore, for difficult search tasks, the number of true-positive detections would be reduced significantly and the number of false-positives would further increase ( Table 1).

**Table 1.** Similarity values falling into activity-relevant ranges.

MACCS		
Activity Class category	Number of Tc values ≥ 0.8	
	Act vs Act	Act vs Rand (10000 compounds)
***Easy***	39178 **(16.0%)**	1613 **(0.005%)**
***Intermediate***	538349 **(5.0%)**	27423 **(0.01%)**
***Difficult***	559442 **(1.2%)**	70057 **(0.01%)**
ECFP4		
Activity Class category	Number of Tc values ≥ 0.3	
	Act vs Act	Act vs Rand (10000 compounds)
***Easy***	93611 **(38.2%)**	8282 **(0.03%)**
***Intermediate***	1041694 **(10.9%)**	104003 **(0.04%)**
***Difficult***	1854822 **(4.1%)**	233943 **(0.05%)**

Reported are the total number of pairwise compound comparisons and percentages of Tc values (bold) falling into activity-relevant ranges of similarity values identified for the MACCS and ECFP4 fingerprints. “Act” stands for active and “Rand” for random.

The percentages of compounds from different categories falling into activity-relevant similarity ranges reported in Table 1 also helped to rationalize the relative performance of fingerprints in benchmark calculations. Such retrospective calculations typically focus on easy or intermediate activity classes (otherwise, mostly “negative” results would be obtained). In benchmark settings, ECFPs are often superior to MACCS and other standard fingerprints. If compound recall rates are determined, which is usually the case, but only possible in retrospective applications, ECFP4 is clearly favored over MACCS, given the much larger percentage of true-positive detections according to Table 1. However, it should also be noted that even for easy activity classes, ECFP4 only detected less than 40% of active compounds at activity-relevant similarity values. Thus, the false-negative rate was high, even more so for MACCS, indicating that the sensitivity of these fingerprints to active compounds in a similarity search scenario is low. This again reflects the fact that structure-activity information is not explicitly used in a fingerprint search.

In a prospective similarity search application, when active compounds are sparse and unknown and source databases are large, it would be more difficult to draw a line between ECFP4 and MACCS, as discussed above. Then, the ability to identify novel hits will much depend on the specific features of active compounds and the capacity of different fingerprints to capture them.

A plus of activity-relevant similarity values, as determined herein, is that they have been derived over many different activity classes and are thus general in nature. As such, they become a characteristic feature of a given fingerprint, although their utility for practical similarity searching is limited.

## Conclusion

In conclusion, in this study, we have addressed the issue of how, from a fundamental point of view, activity similarity might be related to molecular similarity calculated using fingerprints by focusing on systematic compound comparisons involved in similarity searching. The analysis has led to the introduction of activity-relevant similarity values as a characteristic feature of fingerprints of different design, which we consider useful as likelihood estimates. For example, given our ensemble of activity classes, the likelihood that a compound comparison yielded a Tc value of at least 0.8 for MACCS or 0.3 for ECFP4 was much higher for compounds sharing the same activity than randomly selected compounds. It was also much higher than for comparison of active vs. randomly selected compounds.

## Data availability

The data referenced by this article are under copyright with the following copyright statement: Copyright: © 2016 Jasial S et al.

ZENODO: Activity classes from different categories, doi: http://dx.doi.org/10.5281/zenodo.47315 ^28^
