# Supplementary material for: Activity-relevant similarity values for fingerprints and implications for similarity searching
Source: F1000Res. 2016 Apr 28;5:Chem Inf Sci-591. Originally published 2016 Apr 6. [Version 2] doi: 10.12688/f1000research.8357.2 (PMC4830209; doi:10.12688/f1000research.8357.2)
Supplement: Peer review history

# Peer review: Referee response for version 1

**DOI:** 10.5256/f1000research.8986.r13264

**Published:** 2016-04-12

**Authors:** Wendy Warr

**Affiliations:** 1 Wendy Warr & Associates, Holmes Chapel, UK

This is a short but interesting paper and is extremely well written. I use the adjective “short” because the novel results occupy fewer than eight pages, if the figures and tables are ignored. Nevertheless, the results are interesting and well worth publishing because they do address a significant problem. The issue in question is well explained on pages 3-5. The literature background appears to cover all relevant research, but I suggest that two of the references be changed. I would replace the ACS meeting abstract cited at 12 with Brown and Martin, 1997 ^1^. That paper does not mention “85%” specifically, but it does discuss the cutoff threshold in detail. Reference 14 is useless: a researcher novel to the field of similarity could not locate MACCS keys by seeking a non-existent company which had an office in San Leandro in 2005. I would prefer to see “activity classes” written out in full: it is not a long-winded term, and ACs looks a bit like a typo for ACS.

On page 8 the sentence “Thus, similarity searching can be mimicked by systematically comparing compounds having the same activity and active compounds to random database compounds” is not clear enough. Further down the page it is made clear exactly what is compared with what, but that is too late. I also did not fully understand the statement “Comparison of random database compounds is not carried out during similarity searching. However, the similarity value distribution resulting from the latter comparison can be monitored as an additional reference.” Maybe: “Comparison of random database compounds to random database compounds is not carried out during traditional similarity searching, but the similarity value distribution resulting from this comparison can be monitored as an additional reference in mimicked similarity searching”? (Note also that it is better not to start a sentence with “However”.)

On page 13 there should be a heading saying “Conclusion” before the sentence that begins “In conclusion”.

The sentence beginning “In conclusion, in this study, we have addressed the issue how molecular similarity calculated using fingerprints and activity similarity might be related to each other from a fundamental point of view…” is ambiguous, e.g., “…molecular similarity calculated using both fingerprints and activity similarity, might be related to what?” Admittedly there is no comma, but it would be clearer to say “In conclusion, in this study, we have addressed the issue of how, from a fundamental point of view, activity similarity might be related to molecular similarity calculated using fingerprints…” At the very end the phrase “…was hundreds of times higher for compounds sharing the same activity than randomly selected or active vs. random compounds.” is not clear enough.

In short, I like the science, and I think it should be indexed, but I would like to see a few minor improvements to the text as detailed above.

I have read this submission. I believe that I have an appropriate level of expertise to confirm that it is of an acceptable scientific standard.

## References

[1] BrownRMartinY: The Information Content of 2D and 3D Structural Descriptors Relevant to Ligand-Receptor Binding. *Journal of Chemical Information and Computer Sciences*.1997;37(1) : 10.1021/ci960373c 1-9 10.1021/ci960373c

# Peer review: Referee response for version 1

**DOI:** 10.5256/f1000research.8986.r13265

**Published:** 2016-04-12

**Authors:** Peter Ertl

**Affiliations:** 1 Novartis Institutes for Biomedical Research, CH-4056 Basel, Switzerland

An interesting manuscript focusing on a relationship between molecule similarity and biological activity, one of the most important (and still not fully solved) problems of applied cheminformatics. The topic is therefore relevant to drug design.

The question the authors are trying to answer is the significance of similarity thresholds when using MACCS and ECFP4 fingerprints and its implications in virtual screening, when one tries to identify small number of active molecules in the large number of inactives.

There have been several studies focusing on the same question (an influence of a similarity thresholds on discriminating active and inactive molecules). Although such studies are mentioned in the literature overview it would be interesting to directly compare their conclusions with the conclusions of the present paper in the “Implications for similarity searching” section.

The information content of the MACCS keys and the ECFP fingerprints are vastly different. The MACCS keys are, to my knowledge, no more used in a productive set-up as molecule descriptors in discriminating between actives and inactives. It would be interesting to focus on additional, more relevant structure descriptors, for example Daylight-like linear fingerprints or topological torsions. I suggest this as a topic for a follow-up study.

The authors should mention which software they used for the calculation of fingerprints. Did they used PipelinePilot, open source tools or their own software? Results generated by different software tools may differ in some cases considerably, based on different molecule normalization, treatment of aromaticity, tautomers etc..

I recommend to mention also a classical paper from this area by Brown and Martin ^1^.

I have read this submission. I believe that I have an appropriate level of expertise to confirm that it is of an acceptable scientific standard.

## References

[1] BrownRMartinY: The Information Content of 2D and 3D Structural Descriptors Relevant to Ligand-Receptor Binding. *Journal of Chemical Information and Computer Sciences*.1997;37(1) : 10.1021/ci960373c 1-9 10.1021/ci960373c

# Response

**Published:** 2016-04-13

**Authors:** Jürgen Bajorath

**Affiliations:** Department of Life Science Informatics, B-IT and LIMES Institutes, Rheinische Friedrich-Wilhelms-Universität Bonn, Germany

Thank you for suggesting the follow-up investigation. We note that the results of the two most relevant investigations were discussed in the introduction.

# Peer review: Referee response for version 1

**DOI:** 10.5256/f1000research.8986.r13263

**Published:** 2016-04-07

**Authors:** Georgia B. McGaughey

**Affiliations:** 1 Vertex Pharmaceuticals, Boston, USA

The authors summarize in detail the threshold between actives and inactives using 2D fingerprints for the MACCS and ECFP4 fingerprint methods using data derived from ChEMBL. The paper is well written and should be indexed.  A few suggestions are made, however:

 

1. Given that this is a chemistry paper, perhaps a few examples of chemical compounds showing the threshold for an active in Tc and ECFP4 space. How “low” can one go and still have an active? This would bolster the need for chemoinformatic approaches over the medchemists’ view of “eyeing” similarity.
2. You have numerous references and mention belief theory in passing. I couldn’t help but think of Muchmore’s paper ^1^ and think you might want to include this paper as well especially given that he uses MACCS and ECFP4.
3. You make no mention of 3D similarity methods, which even in passing, I recommend you include (ie a reference). I have one in JCIM from 2006 comparing 2D to 3D (but it’s not pairwise).
4. What about the overlap between the methods in terms of actives?  The result begs the question to the reader – do I now compute both and take an average (if I can only screen X%)?
5. The threshold of 10uM for an active seems *extremely* generous. In practice, I would typically consider this an inactive compound especially if the screen was an enzymatic screen. How would the results differ if you used a different active threshold?
6. Besides molecular weight, was there any consideration given to the number of PAINS or REOS flags? By this question I’m trying to understand if “actives” were easier to discriminate if compounds were merely promiscuous and if that mattered based on the easy-intermediate-hard ACs (have to agree with Wendy Warr on this – if you could spell out AC – I kept thinking activity cliffs).

I have read this submission. I believe that I have an appropriate level of expertise to confirm that it is of an acceptable scientific standard.

## References

[1] MuchmoreSWDebeDAMetzJTBrownSPMartinYCHajdukPJ: Application of belief theory to similarity data fusion for use in analog searching and lead hopping. *J Chem Inf Model*.2008;48(5) : 10.1021/ci7004498 941-8 10.1021/ci7004498 18416545

# Response

**Published:** 2016-04-13

**Authors:** Jürgen Bajorath

**Affiliations:** Department of Life Science Informatics, B-IT and LIMES Institutes, Rheinische Friedrich-Wilhelms-Universität Bonn, Germany

It is noted that the Muchmore *et al*. reference was already cited. In addition, 3D similarity measures were not considered herein. By design, the study did neither focus on compound overlap for alternative fingerprint representations nor on general compound liabilities.
